# Psilocybin-containing mushroom-assisted psychotherapy is associated with sustained psychological and somatic improvements: two case studies from Jamaica

**DOI:** 10.3389/fpsyt.2026.1876189

**Published:** 2026-07-29

**Authors:** Caitlyn Hanna Sams, Jasmine Davis, Anuradha Ramdas, Rupika Delgoda, Denise Vidot, Marvin Reid, Winston De La Haye

**Affiliations:** 1 Caribbean Centre for Research in Biosciences, The University of the West Indies Mona Campus, Kingston, Jamaica; 2 Sylvester Comprehensive Cancer Center, The University of Miami, Miami, FL, United States; 3 Department of Community Health and Psychiatry, The University of the West Indies Mona Campus, Kingston, Jamaica

**Keywords:** anxiety, depression, high-dose, Jamaica, microdose, psilocybin, PTSD, supervised session

## Abstract

We report two case studies that examine the therapeutic potential of structured psilocybin-containing mushroom biomass regimens administered within a clinical framework, evaluating their impact on mental health outcomes as well as associated somatic symptoms. Two patients underwent the De La Haye Psilocybin Treatment Protocol (DPTP), which integrates micro- and high-doses (combined psilocybin and psilocin content of 1–2 mg versus 40–60 mg) with psychohistoriographic psychotherapy. Follow-up included an integration session and subsequent office visits, including the completion of the Revised Mystical Experience Questionnaire (MEQ-30) two months post-treatment. Case 1 described a 29-year-old female with social anxiety, self-criticism, chronic musculoskeletal pain, and a recent diagnosis of diabetes. Post-treatment, she reported reduced shame, increased self-confidence, and substantial reductions in pain severity. Case 2 involved a 44-year-old female presenting with depression, hopelessness, and long-COVID-related anosmia and ageusia. She reported enhanced psychological resilience, improved mood, and resolution of diffuse body aches. Following the therapeutic dose, she also reported a 80-90% recovery of smell and taste after three years of dysfunction, post COVID-19 infection. The MEQ-30 scores were both high, indicating profound mystical-type experiences. These cases demonstrate improvements in psychological and somatic symptoms following participation in the DPTP. While the pain reduction and observed recovery of olfactory and gustatory function is noteworthy, spontaneous improvements cannot be excluded and further controlled investigation is warranted.

## Introduction

Mental health disorders represent a major contributor to the global burden of disease, with mental disorders being among the top 10 leading causes of health loss worldwide (1). In 2019, one in every eight people around the world were living with a mental disorder, including anxiety, depressive disorders, and post-traumatic stress disorder (PTSD) among many others (2). In Jamaica, depression and anxiety disorders are among the most common mental health concerns, with approximately 14.3% of Jamaicans suffering from depression and 4.1% from anxiety disorders (3, 4). These rates are rising, particularly among adults aged 20-59 (4). Yet despite the increasing prevalence, access to conventional psychiatric treatment in Jamaica remains limited due to resource constraints, stigma, and the cost of care (5). This treatment gap has fueled interest in conventional and innovative approaches as well as alternative therapies that may help address unmet needs.

One emerging option is the therapeutic use of psilocybin-containing mushrooms, commonly referred to as “magic mushrooms.” These mushrooms biosynthesize a tryptamine alkaloid compound called psilocybin, which is rapidly dephosphorylated in the body to form psilocin, the bioactive compound responsible for altering mood, perception, and cognition (6, 7). Clinical research has demonstrated psilocybin’s potential to reduce symptoms of depression and anxiety, alleviate obsessive-compulsive disorder, and improve outcomes in alcohol and tobacco use disorder (8, 9). Beyond symptom relief, patients often report improvements in quality of life, social connectedness, and psychospiritual well-being following psilocybin therapy. These effects are thought to arise primarily through agonism of the serotonin 5-HT_2A_ receptors in the central nervous system, leading to neuroplastic and psychological changes (10).

Psilocybin use is not new. Archaeological evidence of mushroom rituals in North Africa date back to 6000 BC (11), while in Mesoamerica, ritualistic consumption is well documented through mushroom stones and illustrated codices such as the 14th-century Codex Mexicana (12). In the mid-20th century, ethnobotanist Robert Gordon Wasson’s encounter with Mazatec psilocybin ceremonies in Mexico introduced *Psilocybe* mushrooms to a global audience. Soon after, chemist Albert Hofmann identified psilocybin (4-phosphoryloxy-N,N-dimethyltryptamine) and its dephosphorylated derivative psilocin (4-hydroxy-N,N-dimethyltryptamine) as the active constituents of the mushrooms (7). These discoveries helped lay the foundation for modern scientific inquiry into psilocybin’s pharmacology and therapeutic potential.

Today, psilocybin research has surged, with clinical trials in North America and Europe demonstrating promising results in treatment-resistant depression, end-of-life anxiety, and substance use disorders (8). In Jamaica, the use of natural and traditional therapies has long been culturally accepted and preferred for managing a range of medical conditions (13, 14). Uniquely, Jamaica represents one of the few countries where psilocybin-containing mushrooms are legally available, creating a rare opportunity for scientific research and clinical application (15).

Within this context, Dr. Winston De La Haye has been advancing psilocybin therapy in Jamaica through the De La Haye Psilocybin Treatment Protocol (DPTP) at his private psychiatry practice in Kingston, Jamaica (15). The program utilizes *Psilocybe cubensis* (*P. cubensis*) psilocybin-containing mushrooms cultivated, tested, and formulated at the Good Manufacturing Practice (GMP)-certified Future Wellness Lab of the University of the West Indies. The biomass is prepared as capsules for low-dose administration and chocolate bars for high-dose sessions (16, 17), ensuring standardized psilocybin dosing and delivery under in-patient supervision at Dr. De La Haye’s private practice. To date, over 40 patients have enrolled in the program, with therapeutic progress assessed via clinical examination and tools such as the psychohistoriogram and the Mystical Experience Questionnaire (MEQ-30).

Dr. De La Haye developed the DPTP, which integrates psilocybin-containing mushroom treatment with Psychohistoriographic Brief Psychotherapy, a modality first conceptualized in the 1970s by Professor Frederick Hickling at Bellevue Mental Hospital, Kingston. Psychohistoriography is a Caribbean model of psychotherapy that uses history as the primary tool for promoting psychological insight (15). Patients chart memories and significant life events within a dialectic matrix called the psychohistoriogram throughout the course of the treatment, which includes life domains such as family, education, religion, politics, sexuality, and culture (15, 18).

After the experience, patients completed the MEQ-30, a validated 30-item self-report instrument with strong psychometric reliability for characterizing psilocybin-induced mystical-type experiences (19). The MEQ-30 assesses four core domains (mystical, positive mood, transcendence of time and space, and ineffability) and has been shown to predict sustained changes in attitudes, behavior, and well-being independent of the acute intensity of drug effects (19, 20). Inclusion of the MEQ-30 provided a standardized framework for assessing the subjective and potentially therapeutic dimensions of the psilocybin experience.

In these case studies, two patients who enrolled in the DPTP are examined, with their treatment responses illustrating varying degrees of improvement in health outcomes and motivating a broader discussion of psilocybin’s therapeutic impact. This work contributes to a growing body of evidence by examining the impact of controlled psilocybin-containing mushroom therapy on psychiatric patients in Jamaica. In addition to improvements in mental health symptoms, several patients also reported unanticipated benefits, including relief from chronic pain and improvement of Long COVID-19 symptoms following the eight-week treatment period. By documenting these findings, this study aims to expand the understanding of psilocybin’s therapeutic potential and situate Jamaica as a unique setting for advancing psychedelic research.

## Case studies

### Case 1

This patient was a 29-year-old female who was referred to Dr. Winston De La Haye at his private psychiatry practice in November 2022 for the management of depression, anxiety, self-esteem concerns, and post-traumatic stress disorder (PTSD) consistent with DSM-5-TR diagnostic criteria (21). During the course of her care, she was diagnosed with diabetes mellitus in May 2024 by her family physician. In addition, she reported chronic musculoskeletal pain affecting her left shoulder and left ankle from previous injury due to physical activity, which she described as persistent and interfering with her daily functioning.

At the time of her initial visit, she was taking sertraline (Zoloft) 50 mg by mouth daily for her depression. Over the course of her care, there were intermittent periods of time with discontinuation of her medication. However, she continued with psychological support with a psychologist throughout the entirety of her treatment. Following her diabetes diagnosis in May 2024, she was initiated on metformin (one tablet three times daily) and gliclazide (one tablet each morning) for glycemic management.

The patient had a longstanding psychiatric history, reporting feelings of depression since childhood, when she experienced physical, verbal, and sexual abuse. Her depressive symptoms included sadness, irritability, anhedonia, fatigue, and decreased libido. She was diagnosed with dysthymia and major depressive disorder (MDD), with concurrent eating disorder behaviors characterized by binge eating and self-induced vomiting since the age of twelve. Diagnostic workup included blood tests in March 2024, which later confirmed diabetes in May 2024.

Given minimal improvement from treatment with sertraline, the patient was assessed as an appropriate candidate for the DPTP. All of the details about the treatment regime were explained to the patient, including biomass species, indications, and dosing. The patient confirmed that she had a good understanding of the DPTP and confirmed that she wanted to participate.

### Case 2

This patient was a 44-year-old female referred to Dr. Winston De La Haye at his private psychiatry practice in December 2021 for evaluation of anxiety and insomnia. She was subsequently diagnosed with complex PTSD (PTSD with depressive features), consistent with DSM-5-TR diagnostic criteria (21). In addition to psychiatric symptoms, the patient reported chronic somatic pain. She also experienced anosmia and ageusia following a COVID-19 infection in 2021, a history that was not disclosed to Dr. De La Haye at the time of initial assessment and had persisted without improvement until the time of psilocybin treatment.

Her past medical history was significant for pulmonary embolism, supraventricular tachycardia, and chronic insomnia. She was treated for her heart condition with atenolol initially and later switched to metoprolol 5 mg daily. At the time of her first psychiatric consultation, she was taking alprazolam (Xanax) 0.25 mg twice daily for her anxiety, paroxetine (Paxil) 12.5 mg once daily for her depression, and zopiclone 7.5 mg nightly for insomnia.

The patient reported a longstanding history of depression and anxiety dating back to childhood. She described experiences of physical and verbal abuse and reported difficulty speaking about her trauma. Instead, she preferred to suppress or “forget” memories of her past. Her psychiatric symptoms included sadness, hopelessness, insomnia, low libido, withdrawal, irritability, anxiety, and episodes of suicidal ideation.

Diagnostic evaluation included blood work conducted in March 2023. Her treatment plan incorporated antidepressant therapy, switching from paroxetine to bupropion XL, and adding a trial of amitriptyline for pain management, which was ultimately discontinued. Sleep disturbances were managed with zopiclone 7.5 mg nightly and adjunctive diphenhydramine 50 mg at night.

After initial stabilization with pharmacotherapy, including titration of bupropion XL to 300 mg daily, the patient was evaluated and approved for participation in the DPTP. All of the aspects about the treatment regime were explained to the patient, including psilocybin-containing mushroom biomass species, therapeutic indications, and dosing. The patient demonstrated adequate understanding of the protocol and provided informed consent to participate.

## Materials and methods

### Preparation of treatment agents: *P. cubensis* mushroom biomass

*P. cubensis* mushrooms were cultivated and processed at Future Wellness, a GMP-certified and legally regulated laboratory at the University of the West Indies Mona in Kingston, Jamaica. Fruiting bodies were produced under stringent cultivation conditions, and standard protocols were followed for harvesting, drying, sorting, and milling (16). The milled biomass underwent comprehensive quality-control testing, including high-performance liquid chromatography (HPLC) for potency, and assays for heavy metals, moisture content, mycotoxins, pesticide residues, and microbial contamination (17). Only batches meeting all safety and quality criteria were used for manufacturing.

Mushroom capsules were prepared from analytically characterized powdered *P. cubensis* mushroom biomass using a capsule maker. Each capsule contained mushroom biomass with quantified total combined psilocybin and psilocin content of 1 mg, 3 mg, or 10 mg, consisting predominantly of psilocybin (~90%) with a smaller proportion of psilocin (~10%). These preparations corresponded to approximately 100 mg, 300 mg, or 1 g of dried biomass, respectively. However, because natural mushroom biomass exhibits batch-to-batch variability in alkaloid concentration, dosing was determined based on the analytically measured combined psilocybin and psilocin content rather than biomass weight. Capsules were stored in airtight, opaque containers at room temperature (25°C), protected from light and moisture, and typically distributed to the clinician on the same day (17). Clinicians and patients were instructed to store the capsules in the same stringent conditions.

For chocolate production, *P. cubensis* mushroom biomass was homogenized into dark chocolate under standard preparation conditions to promote even distribution of active compounds, by an expert chocolatier. Each chocolate bar contained 30 mg of combined psilocybin and psilocin, corresponding to approximately 3 g of dried biomass based on prior potency analyses (17). No additional psychoactive substances, medicinal compounds, or other ingredients were included in the formulation. Finished products were similarly stored in airtight, opaque containers at 4°C until use.

### De La Haye psilocybin treatment protocol

**Figure 1:**
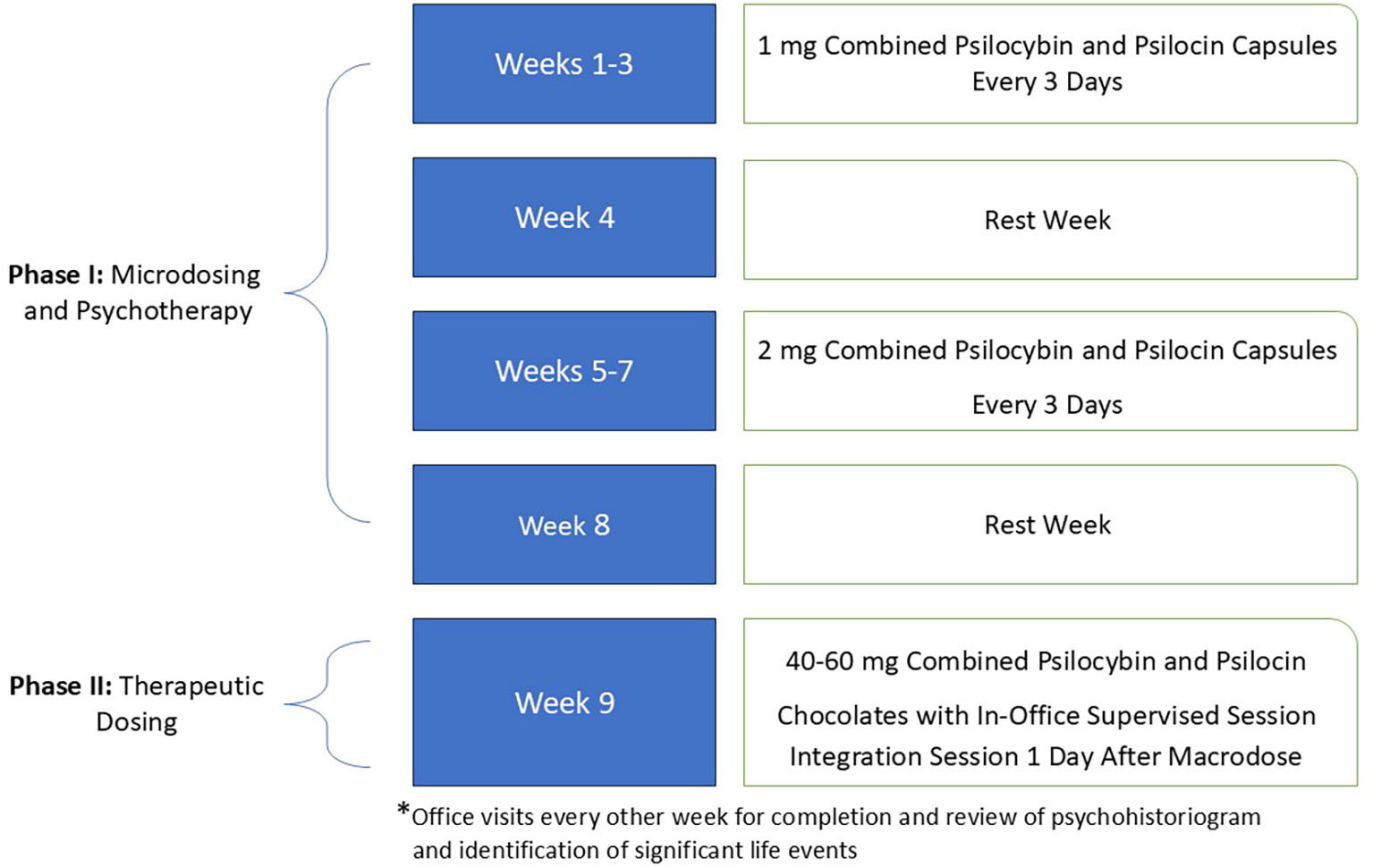
*The De La Haye psilocybin treatment protocol (DPTP).* Schematic overview of the two-phase DPTP, consisting of an 8-week outpatient microdosing and psychotherapy phase followed by an optional supervised therapeutic-dose session in week 9. Psilocybin-containing *P. cubensis* biomass preparations were administered based on analytically quantified total psilocybin and psilocin content (approximately 90% psilocybin and 10% psilocin). Phase I includes incremental microdosing (1–2 mg combined psilocybin and psilocin content every third day) with psychotherapy and psychohistoriogram development, followed by Phase II therapeutic-dose administration under clinical supervision with subsequent integration support.

The DPTP consists of two phases (**Figure 1**):

#### Phase I (microdosing and psychotherapy)

Patients undergo an 8-week outpatient program centered on documenting and reflecting upon their psychohistoriogram. During this phase, patients receive oral microdosing administrations of *P. cubensis* biomass containing 1 mg or 2 mg of combined psilocybin and psilocin every third day at home (with two rest days between doses), alongside ongoing psychotherapy sessions occurring every 2 weeks. The psychotherapy sessions allowed patients to explore personal history, significant life experiences, trauma, emotional patterns, and psychosocial stressors.

During weeks 1-3, patients receive 1capsule containing 1 mg of combined psilocybin and psilocin per administration every three days. Week 4 serves as a medication-free reflection period to allow patients to assess their response and tolerability. During weeks 5-8, dosing increases to 2 mg of combined psilocybin and psilocin every third day (two of the 1 mg capsules). A second 1-week break concludes Phase I, after which the completed psychohistoriogram is reviewed with the patient.

Throughout Phase I, patients continued their prescribed medications as described in the individual case reports. As part of clinical management, medication regimens were reviewed prior to initiation of the DPTP, and both patients underwent physician-directed tapering of their antidepressant medications to half of their baseline dose by the week of the therapeutic-dose session (Phase II). Specifically, Case 1 reduced sertraline from 50 mg daily to 25 mg daily, while Case 2 reduced bupropion XL from 300 mg daily to 150 mg daily prior to the therapeutic-dose session. Patients underwent routine psychiatric and medical assessments before receiving *P. cubensis* mushroom biomass preparations, and potential medication-related risks were considered as part of the clinical evaluation. Patients were monitored throughout treatment for psychological adverse effects. No serious adverse events related to medication interactions were identified in either case. A detailed timeline of concomitant medication use before, during, and after participation in the DPTP, including physician-directed medication changes, is provided in **Appendix A**.

#### Phase II (optional therapeutic dosing)

Patients who elect to continue proceed to an in-office therapeutic-dose session during week 9. The patient in Case 1 received 40 mg of combined psilocybin and psilocin (a 30 mg chocolate preparation plus a 10 mg capsule), while the patient in Case 2 received 60 mg of combined psilocybin and psilocin (two 30 mg chocolate bars).

To reduce potential nausea associated with administration, patients received one tablet of dimenhydrinate (Gravol) prophylactically approximately 30 minutes before breakfast on the day of the therapeutic-dose session.

These supervised sessions are conducted under medical supervision of the treating psychiatrist and a trained psychiatric nurse sitter with concurrent psychotherapeutic support (15). Patients received the therapeutic-dose in a prepared, supportive environment with calm music, eyeshades, and a comfortable reclining position. Additional details regarding the structure and components of the set and settings of the DPTP therapeutic-dose are provided in **Appendix B**.

Following the therapeutic-dose session, both patients elected to discontinue antidepressant medication under the supervision of the treating psychiatrist with ongoing clinical monitoring.

### Monitoring throughout treatment

Integration is a critical component of the DPTP. The morning following therapeutic-dose administration, patients attended an hour-long, in-office integration session in which they revisited and reflected upon their psychohistoriogram under therapist guidance (15). During this session, patients were supported in processing their experiences, identifying personally meaningful themes, and integrating perceived insights into their ongoing therapeutic work. Additionally, the patient in Case 2 submitted a written reflection describing aspects of their experience, including reported mystical-type experiences, which was used to further support the integration process.

Research follow-up monitoring occurred approximately two months after the therapeutic session and coincided with routine clinical follow-up contact. During this follow-up, both a standardized questionnaire and a semi-structured interview were conducted in-person with Case 1 and remotely via telephone with Case 2. The MEQ-30 was administered to assess the subjective quality of the psilocybin experience. The MEQ-30 is a validated 30-item self-report measure widely used in psilocybin research (19). Consistent with prior studies, a total MEQ-30 score >90 was used to classify a “complete mystical experience.” Full details regarding instrument structure, scoring, and psychometric properties are provided in **Appendix C**.

Following completion of the MEQ-30, a semi-structured interview was conducted. The interview was conversational and patient–centered, with prompts tailored to aspects of the experience the participant felt comfortable discussing. This qualitative component provided additional context to complement the quantitative MEQ-30 data and capture patient-reported experiences following treatment.

## Results

No serious adverse events were reported during or following the therapeutic sessions. Patients tolerated the psilocybin-containing mushroom biomass sessions without clinically significant complications. Acute psychological and perceptual effects expected during the acute psychedelic experience were not classified as adverse events in these cases. Routine clinical follow-up did not identify persistent adverse effects related to the intervention.

### Case 1

Prior to participation in the DPTP, this patient reported self-criticism, shame, and social anxiety, particularly related to public speaking. She also reported a recent diagnosis of diabetes and chronic musculoskeletal pain in her left shoulder and left ankle.

During the integration session and follow-up after completion of the DPTP, the patient described marked improvements in psychological well-being, including increased self-confidence, greater comfort with self-expression, and substantial reductions in shame and social anxiety. She expressed increased ease in attending medical appointments and no longer concealed her participation in therapy from her friends or family.

Following sustained clinical improvement, Dr. De La Haye supervised a reduction and discontinuation of her sertraline 25 mg. At her follow-up sessions, she continued to report sustained improvements in psychological well-being.

Somatic outcomes included notable reductions in shoulder and ankle pain, which she attributed to the psychedelic experience, along with heightened bodily awareness and improved coping related to her diabetes diagnosis.

Her MEQ-30 total score was 99, exceeding the established threshold for a complete mystical experience (≥90). This score reflects a moderate-to-high intensity mystical-type experience and supports the clinical relevance of the subjective effects reported following treatment.

### Case 2

Prior to the DPTP treatment, this patient reported persistent symptoms of hopelessness, sadness, and fear of vulnerability. Additionally, she experienced anosmia and ageusia due to Long COVID-19 since 2021.

During the integration session and follow-up, the patient reported substantial improvements in mental health, including renewed hope, increased resilience in managing depression and anxiety, improved sleep, and greater willingness to engage with past trauma. Somatic symptoms, including diffuse body aches and pain, resolved after treatment. She described improved mood, increased positive affect, and enhanced emotional regulation. An additional unanticipated outcome was partial recovery of patient-reported olfactory function. The patient reported an 80-90% return of her sense of smell by May 2024, approximately three years after COVID-19-related anosmia. These clinical improvements were concordant with her MEQ-30 score of 119, indicating a high-intensity, complete mystical experience.

Following sustained clinical improvement, Dr. De La Haye supervised tapering and discontinuation of her antidepressant medication. At her December 2024 follow-up, she had successfully discontinued antidepressant therapy under physician guidance and remained medication-free aside from zopiclone 7.5 mg nightly for sleep support. She reported improved mood, reduced anxiety, and greater engagement in exercise and social activities, with no recurrence of suicidal ideation.

## Discussion

Conventional treatments for depression, anxiety, and post-traumatic stress disorder typically involve combinations of psychosocial interventions, psychotherapy, and pharmacological management (22, 23). Psychosocial approaches range from education, stress management, and structured social reactivation programs to more intensive therapies such as cognitive behavioral therapy, interpersonal therapy, and relaxation training. Pharmacological interventions include antidepressants, anxiolytics, mood stabilizers, and in some cases, antipsychotics or neuromodulation therapies. While these treatments have demonstrated efficacy, a substantial proportion of patients experience limited benefit, adverse effects, or incomplete remission (24, 25). This treatment gap underscores the need for novel therapeutic approaches.

In recent years, psilocybin-assisted therapy has emerged as a promising modality for addressing a wide range of mental health conditions, including depression, anxiety, and trauma-related disorders. Psilocybin facilitates profound alterations in consciousness, often described as mystical-type experiences, which have been shown to correlate with improved clinical outcomes (26).

In the cases presented here, patients demonstrated substantial psychological benefits following high-dose, supervised psilocybin therapy, including reductions in shame, hopelessness, and fear, as well as increased self-confidence, resilience, and acceptance. These subjective reports were supported by their MEQ scores, which confirmed that the patients experienced high-intensity mystical-type states, aligning with prior findings that link therapeutic benefit to the depth of the psychedelic experience (8). However, because this case series was not designated to evaluate causal relationships, these observations cannot determine whether psychological improvements were attributable to the psychedelic experience itself, psychotherapy, expectancy effects, or the combined effects of the broader treatment framework.

The psychotherapeutic components of the DPTP may have played an important role in facilitating the outcomes described in these cases. The psychohistoriogram provided a structured opportunity for patients to examine personal experiences, trauma, and longstanding patterns of thought and behavior. Continued therapeutic support throughout the microdose phase and integration following the therapeutic-dose session may have helped patients process emotionally significant experiences and apply insights from treatment to daily life (15). Therefore, the DPTP should be understood as a multicomponent intervention involving preparation, psychotherapy, psilocybin-containing mushroom biomass administration, and integration rather than a pharmacological intervention alone.

Beyond psychological outcomes, psilocybin therapy in these cases was associated with meaningful somatic improvements. Case 1 reported a reduction in chronic musculoskeletal pain and improved ability to cope with a new diagnosis of diabetes, suggesting that psilocybin may influence the perception of pain and bodily awareness. Prior studies have noted that psychedelics can modulate pain pathways and reduce pain-related distress, making this an area of increasing clinical interest (27, 28).

There are several potential neurobiological mechanisms that could have mediated the analgesic effects observed in Case 1. Psilocybin’s action as a 5-HT2A receptor agonist can modulate cortical and subcortical networks involved in pain perception, including the anterior cingulate cortex, insula, and thalamus, altering the subjective experience of pain (27, 29). Psychedelic-induced neuroplasticity, including enhanced dendritic spine density and synaptogenesis, may contribute to the reorganization of pain-processing circuits, potentially reducing chronic pain signaling (30). Additionally, the profound alterations in consciousness and mystical-type experiences reported by patients can influence affective and cognitive processing of pain, reducing the emotional distress associated with chronic pain (27, 31). Psychedelics may also indirectly alleviate pain by reducing anxiety and depression, which are known to exacerbate pain perception (27, 32). Together, these neurobiological and psychospiritual effects provide a plausible framework for understanding how psilocybin-assisted therapy may lead to reductions in pain.

However, the pain-related observations in this case study should be interpreted cautiously. The study design does not allow determination of whether symptom improvement was related to psilocybin-containing mushroom biomass administration, psychotherapy, changes in mood or anxiety, altered coping strategies, expectancy effects, or natural fluctuations in chronic pain symptoms. Future controlled studies incorporating validated pain measures will be necessary to evaluate whether psychedelic-assisted therapy has clinically meaningful effects on chronic pain.

Case 2 also demonstrated an interesting outcome: partial recovery of olfactory and gustatory function after three years of COVID-19-related anosmia and ageusia. Chemosensory dysfunction has been a well-documented long-term consequence of SARS-CoV-2 infection, with studies estimating that up to 60% of patients lose smell or taste during acute infection and many fail to achieve full recovery (33, 34). Current therapeutic strategies, including corticosteroids, often fail to address persistent symptoms (35).

The mechanisms underlying potential sensory recovery are not fully understood, but may involve neuroplasticity and serotonergic modulation. Among its many different functions, psilocybin acts as a 5-HT2A receptor agonist, which can enhance synaptic connectivity and promote functional reorganization in cortical and subcortical networks (6, 10). In the olfactory and gustatory systems, this could facilitate regeneration or reorganization of neural pathways disrupted by viral infection (36, 37). Additionally, psilocybin-induced increases in neural plasticity and network connectivity may support adaptive sensory processing, allowing previously dormant or impaired pathways to regain function (37, 38).

Although the timing of reported sensory improvement following completion of the DPTP was notable, spontaneous recovery of olfactory and gustatory function can occur even after prolonged periods of dysfunction (39). Additionally, this observation was based solely on patient report, and no objective olfactory testing, gustatory testing, imaging, or biomarkers were obtained before or after treatment. Therefore, the present case cannot establish whether the sensory improvement was related to psilocybin-containing mushroom biomass therapy, and controlled studies with objective assessments are required to investigate this possibility.

The findings of this case series should also be interpreted within the broader context of psychedelic-assisted therapy research. Recent randomized controlled trials and meta-analyses have demonstrated therapeutic potential for psilocybin while also highlighting the importance of appropriate control conditions, blinding, and the influence of expectancy effects. Some studies evaluating lower-dose psilocybin administration have suggested that observed difference between psilocybin and placebo may be reduced when expectancy effects are better controlled (40). However, the degree to which expectancy effects, blinding limitations, and therapeutic context influence outcomes following higher therapeutic-doses of psilocybin remains an active area of investigation (41). The present case series contributes descriptive information regarding implementation of a structured psychedelic-assisted therapy framework in a real-world clinical setting but does not provide evidence of efficacy.

Several limitations should be considered when interpreting these findings. This report describes two individuals receiving routine clinical care and therefore lacks a control or comparison group, limiting the ability to establish causal relationships between the intervention and the reported outcomes. Improvements may reflect expectancy effects, placebo effects, spontaneous symptom variation, concurrent interventions, psychotherapy, or other unmeasured factors. Additionally, standardized baseline and follow-up outcome measures, including validated psychiatric and pain scales, were not collected because these patients were not enrolled in a prospective research protocol. As a result, symptom changes were primarily assessed through clinical observations and patient-reported experiences. Future prospective evaluations of the DPTP should incorporate validated outcome measures collected at multiple time points throughout treatment.

The retrospective administration of the MEQ-30 approximately two months after the therapeutic-dose session represents another limitation. Although MEQ-30 is widely used in psychedelic research, it is typically administered within days of the psychedelic experience to minimize recall bias. Therefore, the scores reported here should be interpreted as retrospective descriptions of subjective experiences rather than definitive assessments of mystical-type experience intensity.

Because the DPTP incorporates multiple integrated components, including psychotherapy, a microdosing phase, therapeutic-dose administration, and post-session integration, the contribution of individual components cannot be determined. This case series was not designed to distinguish whether outcomes emerged from the microdose phase, the therapeutic-dose session, psychotherapy, integration, or their combined effects. Additionally, adherence during the outpatient microdosing phase was based on patient report and could not be independently verified. Future studies should incorporate prospective monitoring strategies to better evaluate treatment adherence and the contribution of individual protocol components.

Both patients continued prescribed medications during treatment, including psychotropic medications with potential serotonergic activity. Although no clinically significant adverse effects suggestive of medication-related complications were observed, the safety and efficacy of psilocybin-containing mushroom biomass therapy in combination with antidepressants and other psychiatric medications remain incompletely understood (42). Future studies should incorporate standardized medication management protocols and prospective safety monitoring.

Taken together, these cases provide preliminary and exploratory observations regarding the feasibility of integrating a structured psilocybin-containing mushroom biomass-assisted therapy framework into clinical practice. The reported psychological and somatic improvements highlighted areas for further investigation while emphasizing the need for larger, controlled studies to evaluate efficacy, safety, mechanisms of action, and the contribution of individual treatment components.

## Conclusion

These cases demonstrate the potential of standardized psilocybin-containing mushroom-assisted psychotherapy, delivered through the De La Haye Psilocybin Treatment Protocol, to alleviate psychological distress and improve somatic symptoms. One patient reported meaningful reductions in anxiety, depression, and somatic pain, while the other patient experienced improved psychological well-being and partial recovery of olfactory and gustatory function after prolonged dysfunction related to long COVID-19. While preliminary, these findings highlight the multidimensional therapeutic potential of psilocybin and the value of integrating structured psychedelic treatment with culturally grounded psychotherapeutic approaches. Further controlled studies are needed to validate these observations, clarify mechanisms of action, and determine the broader clinical applicability to psilocybin-assisted interventions in both mental health, addiction, and somatic domains.

## Data Availability

The original contributions presented in the study are included in the article/**Supplementary Material**. Further inquiries can be directed to the corresponding author.

## Appendices

### Appendix B Set and setting of psilocybin macro-dose supervised session.

In psychedelic-assisted therapy, “setting” refers to the physical, interpersonal, and environmental context in which the psychedelic experience occurs. The therapeutic setting is considered an important component of psychedelic-assisted interventions and may influence patients’ emotional responses, perceptions, and ability to process and integrate aspects of the experience (43, 44). Within the DPTP, the therapeutic-dose session environment is intentionally structured to provide a calm, private, and supportive setting for supervised administration of psilocybin-containing mushroom biomass.

The therapeutic-dose sessions are conducted within the private psychiatric practice. However, the clinical environment is modified for the duration of the session to provide a setting distinct from a typical medical appointment. The practice is closed to routine clinical activities, and only the patient, psychiatrist, trained psychiatric nurse serving as the sitter, and administrative staff member located in an adjacent area are present. The session takes place in a temperature-controlled room arranged to provide a comfortable and minimally distracting environment. The room contains artwork, a television positioned at the patient’s eye level, and a recliner where the patient remains throughout the session. Blankets are available for comfort, and a small table for water is positioned within the room out of the patients’ reach. Separate seating is present for the psychiatrist and sitter. A reading lamp is available behind the recliner to allow patients to write or reflect if desired, typically during the later portion of the approximately six-hour session. The patient is continuously accompanied and is not left alone during the supervised session.

Following ingestion of the therapeutic dose, patients are initially provided with approximately 15 minutes to engage with nature videos displayed on the television while listening to music from a DPTP-selected playlist. Patients are then encouraged to recline comfortably and use eyeshades while continuing to listen to music through headphones. Lighting in the room is reduced to create a calm environment. During the acute psychedelic experience, the psychiatrist and sitter remain present, observing the patient’s wellbeing, documenting relevant observations, and providing support when requested, including assistance with routine needs such as bathroom breaks.

As part of the preparatory phase of the DPTP, patients are introduced to the therapeutic-dose session environment prior to administration. During this preparation period, patients are able to become familiar with the physical setting, including the recliner, lighting conditions, and presence of supervising clinician and sitting. This preparation is intended to reduce unfamiliarity with the environment and support patient comfort during the subsequent supervised therapeutic-dose session.

### Appendix C Mystical experience questionnaire (MEQ-30).

The 30-item revised Mystical Experience Questionnaire (MEQ-30) is a validated self-report instrument designed to assess the intensity and phenomenological characteristics of mystical-type experiences. It is most commonly administered following psychedelic experiences and has been shown to predict persisting changes in attitudes, behaviors, and well-being, particularly in studies involving psilocybin (19).

The MEQ-30 consists of 30 items and typically requires approximately 5 minutes to complete. In this study, the questionnaire was administered retrospectively following the experimental session, consistent with prior validation and clinical trial protocols.

Items load onto four empirically derived factors:

1. Mystical (e.g., internal or external unity, noetic quality, and sacredness)
2. Positive Mood (e.g., joy peace, and love)
3. Transcendence of Time and Space (loss of usual time and space perception, sense of infinity)
4. Ineffability (difficulty describing the experience in words)

Participants rate each item on a 6-point Likert scale ranging from

0 = None (not at all)
1 = So slight, cannot decide
2 = Slight
3 = Moderate
4 = Strong (equivalent in degree to any previous strong experience or expectation of this description)
5 = Extreme (more than ever before in my life and stronger than 4)

Total MEQ-30 scores range from 0 to 150, with higher scores indicating a more intense mystical-type experience. Consistent with prior validation studies, a total score of >90 (60% of the maximum possible score) is used as the threshold for a “complete mystical experience.”

The MEQ-30 has demonstrated strong psychometric properties. Validation studies report high sensitivity (hit rate = 0.95), a false alarm rate of 0.14, overall classification accuracy of 90.2%, and a signal detection sensitivity (d’) of 3.46, supporting its reliability and discriminative validity in experimental psychedelic research. The survey is publically available: https://psychology-tools.com/test/meq-30.

Questions:

1. Freedom from the limitations of your personal self and feeling a unity or bond with what was felt to be greater than your personal self.
2. Experience of pure being and pure awareness (beyond the world of sense impressions).
3. Experience of oneness in relation to an “inner world” within.
4. Experience of the fusion of your personal self into a larger whole.
5. Experience of unity with ultimate reality.
6. Feeling that you experienced eternity or infinity.
7. Experience of oneness or unity with objects and/or persons perceived in your surroundings.
8. Experience of the insight that “all is One.”
9. Awareness of the life or living presence in all things.
10. Gain of insightful knowledge experienced at an intuitive level.
11. Certainty of encounter with ultimate reality (in the sense of being able to “know” and “see” what is really real at some point during your experience.
12. You are convinced now, as you look back on your experience, that in it you encountered ultimate reality (i.e., that you “knew” and “saw” what was really real).
13. Sense of being at a spiritual height.
14. Sense of reverence.
15. Feeling that you experienced something profoundly sacred and holy.
16. Experience of amazement.
17. Feelings of tenderness and gentleness.
18. Feelings of peace and tranquility.
19. Experience of ecstasy.
20. Sense of awe or awesomeness.
21. Feelings of joy.
22. Loss of your usual sense of time.
23. Loss of your usual sense of space.
24. Loss of usual awareness of where you were.
25. Sense of being “outside of” time, beyond past and future.
26. Being in a realm with no space boundaries.
27. Experience of timelessness.
28. Sense that the experience cannot be described adequately in words.
29. Feeling that you could not do justice to your experience by describing it in words.
30. Feeling that it would be difficult to communicate your own experience to others who have not had similar experiences.
