# Supplementary material for: Psilocybin-containing mushroom-assisted psychotherapy is associated with sustained psychological and somatic improvements: two case studies from Jamaica
Source: Front Psychiatry. 2026 Jul 29;17:1876189. doi: 10.3389/fpsyt.2026.1876189 (PMC13461598; doi:10.3389/fpsyt.2026.1876189)
Supplement: Supplementary file 1 [file DataSheet1.pdf]

Appendix A. Patient Medication Timeline

Case 1

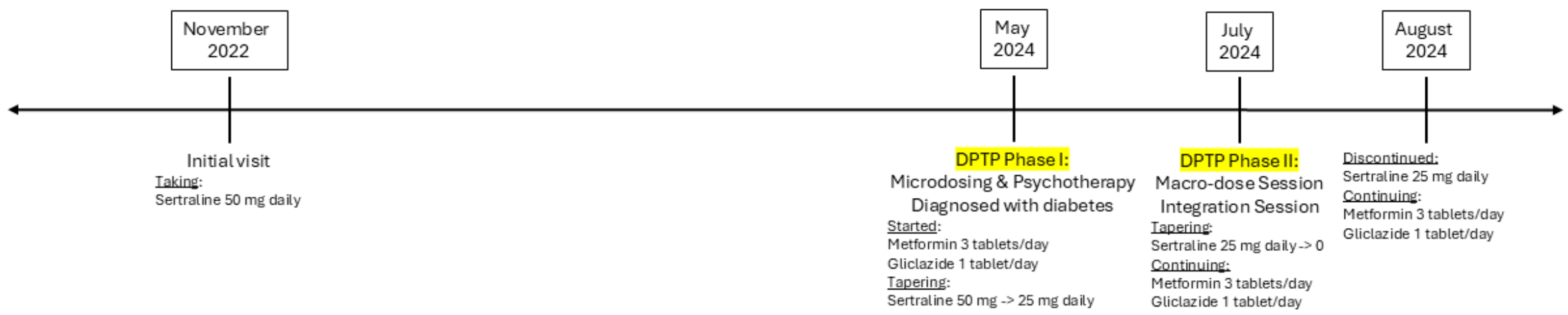

Case 2

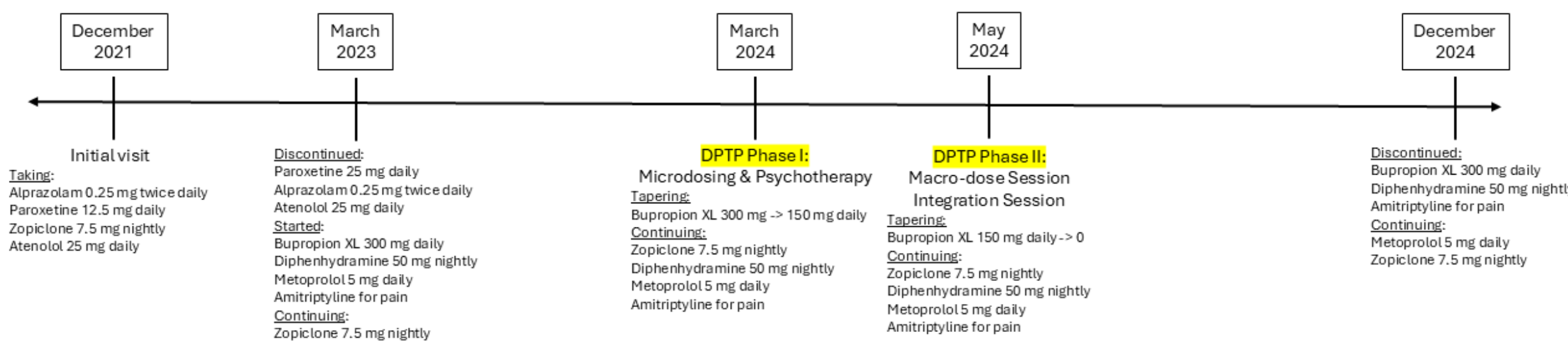

Appendix B. Set and Setting of Psilocybin Macro-Dose Supervised Session

In psychedelic-assisted therapy, "setting" refers to the physical, interpersonal, and environmental context in which the psychedelic experience occurs. The therapeutic setting is considered an important component of psychedelic-assisted interventions and may influence patients' emotional responses, perceptions, and ability to process and integrate aspects of the experience (43,44). Within the DPTP, the therapeutic-dose session environment is intentionally structured to provide a calm, private, and supportive setting for supervised administration of psilocybin-containing mushroom biomass.

The therapeutic-dose sessions are conducted within the private psychiatric practice. However, the clinical environment is modified for the duration of the session to provide a setting distinct from a typical medical appointment. The practice is closed to routine clinical activities, and only the patient, psychiatrist, trained psychiatric nurse serving as the sitter, and administrative staff member located in an adjacent area are present. The session takes place in a temperature-controlled room arranged to provide a comfortable and minimally distracting environment. The room contains artwork, a television positioned at the patient's eye level, and a recliner where the patient remains throughout the session. Blankets are available for comfort, and a small table for water is positioned within the room out of the patients' reach. Separate seating is present for the psychiatrist and sitter. A reading lamp is available behind the recliner to allow patients to write or reflect if desired, typically during the later portion of the approximately six-hour session. The patient is continuously accompanied and is not left alone during the supervised session.

Following ingestion of the therapeutic dose, patients are initially provided with approximately 15 minutes to engage with nature videos displayed on the television while listening to music from a DPTP-selected playlist. Patients are then encouraged to recline comfortably and use eyeshades while continuing to listen to music through headphones. Lighing in the room is reduced to create a calm environment. During the acute psychedelic experience, the psychiatrist and sitter remain present, observing the patient's wellbeing, documenting relevant observations, and providing support when requested, including assistance with routine needs such as bathroom breaks.

As part of the preparatory phase of the DPTP, patients are introduced to the therapeutic-dose session environment prior to administration. During this preparation period, patients are able to become familiar with the physical setting, includign the recliner, lighting conditions, and presence of supervising clinician and sitting. This preparation is intended to reduce unfamiliarity with the environment and support patient comfort during the subsequent supervised therapeutic-dose session.

Appendix C. Mystical Experience Questionnaire (MEQ-30)
The 30-item revised Mystical Experience Questionnaire (MEQ-30) is a validated self-report instrument designed to assess the intensity and phenomenological characteristics of mystical-type experiences. It is most commonly administered following psychedelic experiences and has been shown to predict persisting changes in attitudes, behaviors, and well-being, particularly in studies involving psilocybin (Barrett et al., 2015).
The MEQ-30 consists of 30 items and typically requires approximately 5 minutes to complete. In this study, the questionnaire was administered retrospectively following the experimental session, consistent with prior validation and clinical trial protocols.
Items load onto four empirically derived factors:

1. Mystical (e.g., internal or external unity, noetic quality, and sacredness)
2. Positive Mood (e.g., joy peace, and love)
3. Transcendence of Time and Space (loss of usual time and space perception, sense of infinity)
4. Ineffability (difficulty describing the experience in words)

Participants rate each item on a 6-point Likert scale ranging from

0 = None (not at all)
1 = So slight, cannot decide
2 = Slight
3 = Moderate
4 = Strong (equivalent in degree to any previous strong experience or expectation of this description)
5 = Extreme (more than ever before in my life and stronger than 4)

Total MEQ-30 scores range from 0 to 150, with higher scores indicating a more intense mystical-type experience. Consistent with prior validation studies, a total score of $\geq$90 (60% of the maximum possible score) is used as the threshold for a "complete mystical experience."
The MEQ-30 has demonstrated strong psychometric properties. Validation studies report high sensitivity (hit rate = 0.95), a false alarm rate of 0.14, overall classification accuracy of 90.2%, and a signal detection sensitivity (d') of 3.46, supporting its reliability and discriminative validity in experimental psychedelic research. The survey is publicly available: https://psychology-tools.com/test/meq-30.

Questions:

1. Freedom from the limitations of your personal self and feeling a unity or bond with what was felt to be greater than your personal self.
2. Experience of pure being and pure awareness (beyond the world of sense impressions).
3. Experience of oneness in relation to an "inner world" within.
4. Experience of the fusion of your personal self into a larger whole.
5. Experience of unity with ultimate reality.
6. Feeling that you experienced eternity or infinity.

7. Experience of oneness or unity with objects and/or persons perceived in your surroundings.
8. Experience of the insight that “all is One.”
9. Awareness of the life or living presence in all things.
10. Gain of insightful knowledge experienced at an intuitive level.
11. Certainty of encounter with ultimate reality (in the sense of being able to “know” and “see” what is really real at some point during your experience.
12. You are convinced now, as you look back on your experience, that in it you encountered ultimate reality (i.e., that you “knew” and “saw” what was really real).
13. Sense of being at a spiritual height.
14. Sense of reverence.
15. Feeling that you experienced something profoundly sacred and holy.
16. Experience of amazement.
17. Feelings of tenderness and gentleness.
18. Feelings of peace and tranquility.
19. Experience of ecstasy.
20. Sense of awe or awesomeness.
21. Feelings of joy.
22. Loss of your usual sense of time.
23. Loss of your usual sense of space.
24. Loss of usual awareness of where you were.
25. Sense of being “outside of” time, beyond past and future.
26. Being in a realm with no space boundaries.
27. Experience of timelessness.
28. Sense that the experience cannot be described adequately in words.
29. Feeling that you could not do justice to your experience by describing it in words.
30. Feeling that it would be difficult to communicate your own experience to others who have not had similar experiences.
